# Role of Janus Kinase Inhibitors in Therapy of Psoriasis

**DOI:** 10.3390/jcm10194307

**Published:** 2021-09-22

**Authors:** Sylwia Słuczanowska-Głąbowska, Anna Ziegler-Krawczyk, Kamila Szumilas, Andrzej Pawlik

**Affiliations:** Department of Physiology, Pomeranian Medical University in Szczecin, 70-111 Szczecin, Poland; sylwia@pum.edu.pl (S.S.-G.); ania.ziegler@op.pl (A.Z.-K.); kamila.szumilas@pum.edu.pl (K.S.)

**Keywords:** psoriasis, Janus kinases, therapy

## Abstract

Janus kinases inhibitors are molecules that target Janus kinases—signal transducers and activators of transcription (JAK/STAT). They inhibit this intracellular signal pathway, blocking the gene transcription of crucial proinflammatory cytokines that play a central role in the pathogenesis of many inflammatory and autoimmune diseases, including psoriasis. This process reduces psoriatic inflammation. The JAK inhibitors are divided into two generations. The first generation of JAK inhibitors blocks two or more different Janus kinases. The second generation is more specified and blocks only one type of Janus kinase and has less side effects than the first generation. Tofacitinib, ruxolitinib and baricitinib belong to first generation JAK inhibitors and decernotinib and filgotinib belong to second group. This narrative review summarizes the role of Janus kinase inhibitors in the therapy of psoriasis. Oral JAK inhibitors show promise for efficacy and safety in the treatment of psoriasis. Studies to date do not indicate that JAK inhibitors are superior to recent biologic drugs in terms of efficacy. However, JAK inhibitors, due to their lack of increased incidence of side effects compared to other biologic drugs, can be included in the psoriasis treatment algorithm because they are orally taken. Nevertheless, further studies are needed to evaluate long-term treatment effects with these drugs.

## 1. Introduction

Psoriasis vulgaris is a common inflammatory, chronic skin disease that affects 2% to 3% of the world population. It is a disease with periods of exacerbation and remission. Psoriasis vulgaris has a genetic basis and multigenetic inheritance. Many factors play a role in the development of psoriasis, among which are distinguished: environmental and immunological factors. However, the influence of genetic conditions and multigene background is underlined.

There are two types of psoriasis. Type I is associated with autosomal dominant inheritance, occurring in up to 40 years of age and is associated with HLA-Cw6 tissue compatibility antigens, as well as B13 and B57. Type II appears for the first time between 50 and 70 years of age and is associated with HLA antigens Cw6, Cw2 and B27. Thus far, no specific gene responsible for psoriasis has been found, and HLA-Cw6 alleles are also found in the normal population [1,2,3].

The most common variant of this disease, affecting 85–90% of patients, is plaque psoriasis. In addition, there is palmoplantar psoriasis, erythrodermic psoriasis, and inverse psoriasis as well as generalized pustular psoriasis, which is alternatively termed von Zumbush type. In addition to isolated skin lesions, 25% of patients with psoriasis and joint lesions are diagnosed with psoriatic arthritis [1].

The skin lesions of psoriasis are erythematous scaly plaques, which are preferentially disposed at extensor sites and in areas of mechanic stress such as the knees and elbows. They are characterized by hyperplasia and parakeratosis with accumulation of inflammatory cells in the dermis. In addition, scalp, nails and inverse regions can also be affected [4].

The inflammatory response in psoriasis is mainly driven by T cells, especially T helper cells (Th17), and is mediated by different cytokines, especially TNF-α, IL-17, IL-23 but also other cytokines such as IFN-γ, IL-2, IL-6, IL-8, IL-17, IL-18 and IL-22. The IL-23 is crucial in the pathogenesis of psoriasis and causes Th17 cells to produce IL-17 and IL-22. They induce changes in the skin characteristic for psoriasis. Psoriasis severity is generally characterized by the Psoriasis Area and Severity Index (PASI), which is usually presented as a percentage response rate [2,4,5]. 

There is a wide range of treatment possibilities for psoriasis. The treatments include mainly topical medicines such as ointments with urea, salicylic acid and cygnoline, glucocorticosteroids and vitamin D derivatives and phototherapy. In moderate to severe cases of psoriasis, oral drugs such as acitretin and immunosuppressive drugs such as methotrexate and cyclosporine were given. In recent years, new groups of medicine were used in the treatment of psoriasis, which are biologics. The biologic drugs targeting TNF, IL-12/IL-23, and IL-17 have been approved for the treatment of psoriasis in the last few years, but not all patients respond to treatment with biologics. The biologics are efficient, well tolerated, and safe for treatment of psoriasis but are expensive [4,6,7,8]. The Janus kinase (JAK) inhibitors are a new class of drugs that can be used in systemic treatment of psoriasis, and they are less expensive. 

### 1.1. Janus Kinases

Janus kinase (JAK) is the non-receptor tyrosine kinase that transduces signals from multitudes of cytokines and growth factors and plays a major role in the pathogenesis of many inflammatory and autoimmune diseases, including psoriasis [4,9]. The JAKs are intracellular enzymes that bind to the cytoplasmic domains of cytokine receptors [10,11]. In recent years, there have been many trials about modulating the key intracellular components of cytokine signaling through Janus kinases (JAK) [2,4,12]. 

Cytokines are a group of proteins consisting of different structures. They act on different signal transductions, as a result of joining receptors, and they are grouped depending on the receptor to which they join. The binding of cytokines to their receptors initiates an inflammatory signal that can be mediated by JAK. The large group of cytokines such as IL-2, IL-4, IL-6, IL-7, IL-9, IL-12, IL-15, IL-21, IL-22 and IL23 as well as interferons such as INF-gamma bind to type I and II cytokine receptors [13,14]. 

**Figure 1:**
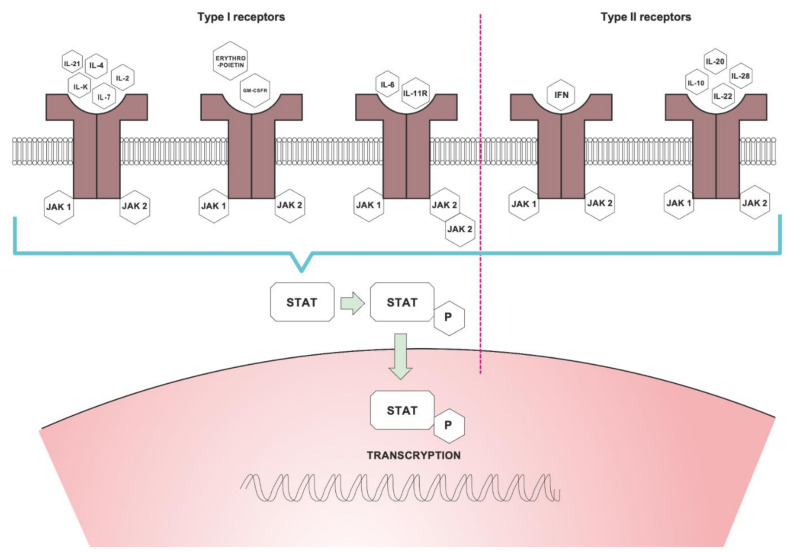
Mechanisms of action of Janus kinases. JAK—Janus kinase, STAT—signal transducer and activator of transcription; P—phosphoric acid, GM-CSF—Granulocyte-macrophage colony-stimulating factor, IFN—Interferon.

When cytokines bind to receptors, the intracellular JAKs are recruited and joined in pairs to the intracellular part of the cytokine receptors, and then, they are activated. The dimerization of JAKs formats heterodimers, autophosphorylate, and attracts STAT (signal transducer and activator of transcription) protein. Afterward, the activated STAT proteins dimerize and translocate to the cell nucleus, where they regulate gene transcription of different cytokines, including proinflammatory cytokines that play role in pathogenesis of psoriasis [6,14,15,16,17] (Figure 1).

JAK was discovered in the end of the last century [18]. In mammals, there are four JAK proteins: JAK1, JAK2, JAK3, and TYK2 (tyrosine kinase 2) [11] and seven STATs [4,11]. JAK1, JAK2, and TYK2 are involved in cell growth processes in different cell types, they partake in their development and differentiation, while JAK3 is critical to hematopoiesis [14,15,19,20]. JAKs are crucial for intracellular signaling of lymphocytes. Their dysfunction is involved with impairment of immune cells [15,21]. The JAK/STAT signaling pathway is typically found in many inflammatory skin diseases including psoriasis [10,13]. It was shown that JAK1 expression correlates with duration of psoriasis and Psoriasis Area and Severity Index (PASI) score [7].

Different JAKs are associated with specific cytokine receptors and influence different aspects of immune cell development and function. JAK1 is associated with INF, IL-6 and Il-10 receptors and with receptors containing the common gamma chain during JAK2 with hematopoetic receptors as well as the IL-12 and IL-23 receptors. JAK3 is associated with major cytokines for lymphocyte function IL-2, IL-4, IL-7, IL-9, IL-15 and IL-21 receptors. The TYK2 is conjuncted with JAK2 and associated with INF, IL-12 and IL-23 receptors [17,21,22]. Mutations of JAK cause dysfunction of cells and diseases such as essential thrombocytopenia, myelofibrosis, polycythemia vera, severe combined immunodeficiency, autoimmune diseases and others [14,16,20,23].

### 1.2. Janus Kinase Inhibitors 

**Figure 2:**
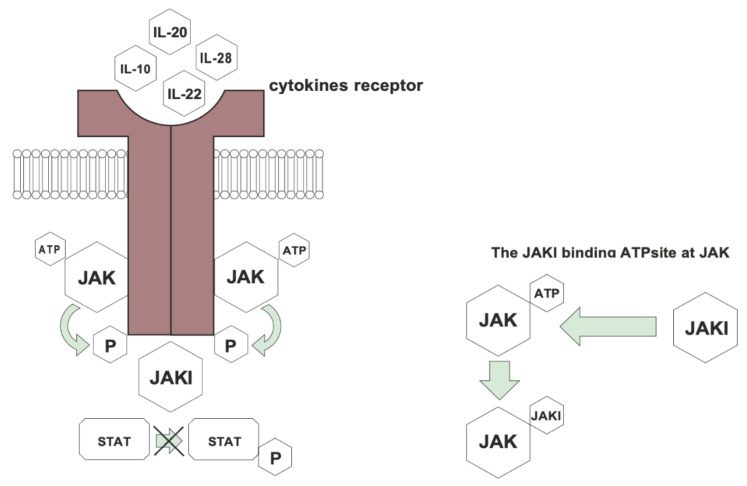
Mechanisms of action of Janus kinase inhibitors. JAK—Janus kinase, JAKI—Janus kinase inhibitor, STAT—signal transducer and activator of transcription; P—phosphoric acid, ATP—Adenosine triphosphate.

JAK inhibitors improve the treatment of many inflammatory diseases, including psoriasis [18]. JAK inhibitors are the molecules targeting the Janus kinase—a signal transducer and activator of transcription (JAK/STAT). They block this intracellular signal pathway by blocking the gene transcription of crucial proinflammatory cytokines, which play a central role in the pathogenesis of many inflammatory and autoimmune diseases including psoriasis [9,10] (Figure 2). This process reduces psoriatic inflammation [14,16,23]. JAK inhibitors target JAKs inside the cell [14,24]. The JAK inhibitors are divided into two generations. The first generation of JAK inhibitors target two or more different JAKs. The second generation is more specified and target only one type of JAK and has less side effects than the first generation [14,25]. Tofacitinib, ruxolitinib and baricitinib belong to first generation of JAK inhibitors and the decernotinib and filgotinib to the second group [13,14,25].

### 1.3. JAK Inhibitors in Psoriasis Treatment

Knowledge about biologics used for psoriasis (such as ustekinumab, secukinumab, ixekizumab, risankizumab) targeting the IL23/IL17 axis, shows that there is also therapeutical potential of JAK inhibitors associated with receptors for these cytokines. The blocking by JAK inhibitors of cytokines pathway may suppress the expression of many cytokines important for pathogenesis of psoriasis [4,14,25,26]. For example, IL-23, the crucial interleukin in the pathogenesis of psoriasis, transduces the signal by JAK2 and TYK2 [14,27] and can be a target for the treatment of psoriasis [4]. 

The JAK inhibitors are currently under clinical investigation for oral and topical treatment in psoriasis [4,10,13,28]. Currently, the three JAK inhibitors, tofacitinib, baricitinib, and ruxolitinib, have been approved for clinical use in psoriasis in the United States of America and Europe [4,29].

### 1.4. Tofacitinib—General Information and Clinical Trials

Tofacitinib is the most studied JAK inhibitor in cutaneous diseases. It is now being explored in skin diseases and do not respond to or sustain intolerable adverse effects as an immunosuppressive and biologic treatment [10,11]. Compared to immunosuppressives and biologics treatment, tofacitinib is easy to administer and can be used orally or topically [11]. Besides being used in psoriasis [4,29], tofacitinib is being used as an off-label indication in alopecia areata, vitiligo and atopic dermatitis [11,15,30]. It is also used in treatment in skin diseases such as moderate to severe active rheumatoid arthritis [15,31,32,33,34], psoriatic arthritis [15,32,35], and ulcerative colitis [15,36].

Tofacitinib, a first-generation JAK inhibitor, blocks tyrosine kinases of the Janus family such as JAK1 and JAK3, with affinity for JAK2 and TYK 2 [10,15,37]. Tofacitinib is rapidly eliminated. The peak level of tofacitinib occurs within 30 min, and the half-life is 3 h. It is metabolized mainly by the liver, primarily mediated by CYP3A4 with minor contribution from CYP2C19, and metabolized at a low percentage in kidneys. It is excreted renally [11,31,38,39,40]. In pregnancy, is not well established and can be used only if benefits outweigh the potential risks. There is a reported small amount of cases of pregnant women who received tofacitinib [11,41]. Tofacitinib is secreted in breast milk and breastfeeding is avoided during treatment [11]. In the pediatric population, studies are not robust. This drug cannot be used in those less than 18 years of age [11]. 

Tofacitinib is the most studied JAK inhibitor used to heal chronic plaque psoriasis orally [14,19,23]. It was shown that treatment with tofacitinib (10 mg twice daily) decreases epidermal thickness, reduces of the number of T cells infiltrating the skin, and suppresses the IL-23/Th17 pathway [11]. 

The action of this drug is decreased during concurrent administration of the potent CYP3A4 inducers (e.g., Rifampicin) and is increased during concurrent administration of potent inhibitors of CYP3A4 (e.g., ketoconazole and flukonazole). The immunosuppressive drugs, e.g., azathioprine, tacrolimus, and cyclosporine, are avoided during treatment with tofacitinib because of increased risk of immunosuppression. In addition, disease-modifying anti-rheumatic drugs and biologics are not well studied and are not recommended for co-administration because of an increased risk of immunosuppression [11]. The therapy with tofacitinib should not be started in the following conditions: active infection, hematological abnormalities, severe hepatic impartment, and hypersensitivity to the active substance or to any excipients [11].

The effectiveness of oral administration of tofacitinib was confirmed in the treatment of moderate to severe plaque psoriasis phase III trials [42,43]. The effectiveness and safety of tofacitinib (in dose 5 and 10 mg twice daily) was described in two phase III trials in patients with active psoriasis arthritis. In these trials, tofacinitib was used in combination with methotrexate, sulfasalazine and leflunomide [33,34,44].

Tofacitinib was also be used as a topical treatment. The topical application of 2% tofacitinib ointment decreased possible systemic adverse effects. It was tested in a phase II trial. It was observed to have a better effect than placebo [24,45].

#### 1.4.1. PIVOTAL 1 and PIVOTAL 2—Phase III Studies of Tofacitinib Treatment 

The most important studies of tofacitinib were Pivotal 1 and 2. The duration of these trials was 52 weeks. These were phase III double-blinded studies, which compared tofacitinib 5 mg twice daily and 10 mg twice daily with placebo. The Pivotal 1 study was conducted in 74 centers and the Pivotal 2 study in 94 centers, both in the USA, Canada, Colombia, Germany, Hungary, Japan (Pivotal 1 only), Mexico, Poland, Puerto Rico (Pivotal 2 only), Serbia, Taiwan and Ukraine. Inclusion criteria was age over 18 years, diagnosis of plaque-type psoriasis for over 12 months before the first dose of tofacitinib, Psoriasis Area and Severity Index (PASI) score over 12, psoriatic lesion involvement greater than 10% body surface area (BSA) and Physician’s Global Assessment (PGA) score of 3 (moderate) or 4 (severe). PGA is a five-point scale that shows global consideration of erythema, induration, and scaling of psoriatic lesions. Patients had to be candidates for systemic therapy or phototherapy independently of use of prior systemic agents. Exclusion criteria: nonplaque psoriasis systemic, infections, evidence of active, latent or improperly treated Mycobacterium tuberculosis infection, present drug-induced psoriasis, malignancy or history of malignancies, and receiving of efalizumab previously [46]. Patients were recruited by the investigators and were randomized 2:2:1 to administer tofacitinib: 5 mg—745 patients, 10 mg—741 patients or placebo—373 patients, twice daily. 

End points consisted of the proportion of patients achieving PASI 90 at week 16, the percentage change from baseline in BSA at week 16, change from baseline Dermatology Life Quality Index (DLQI) total score at week 16, the proportion of patients achieving PGA response at week 4, change from baseline DLQI total score at week 4, the proportion of patients achieving PASI 75 at week 4, and percentage change from baseline Nail Psoriasis Severity Index (NAPSI) at week 16 in patients with nail psoriasis at baseline. Another secondary efficacy end point included time to PASI 75 or PGA response to week 16. Patients who received placebo were randomized at week 16 to be given tofacitinib 5 or 10 mg twice daily—it continued until week 52. Patients who did not achieve PASI 75 or PGA score of “clear” or “almost clear” at week 28 were drawn back [42,43].

In this study, it was observed during Pivotal 1 and Pivotal 2, with similar protocols, that the efficacy of oral tofacitinib, with the 10 mg twice daily, was more efficacious than the 5 mg daily. The psoriasis patients who received tofacitinib in 5 or 10 mg twice daily achieved PASI75 at week 16 in higher percentages (OPT Pivotal 1, 5 mg: 39.9%; 10 mg: 59.2% and OPT Pivotal 2, 5 mg: 46.0%; 10 mg: 59.6%), compared with those receiving placebo (OPT PIVOTAL 1: 6.2%; OPT PIVOTAL 2: 11.4%). The proportions of patients achieving PGA responses at week 16 with tofacitinib 5 and 10 mg twice daily were in OPT Pivotal 1: 41.9% and 59.2% versus placebo 9.0%, and in OPT PIVOTAL 2: 46.0% and 59.1% versus placebo 10.9%. These results were maintained until month 24. Discontinuation of treatment by tofacitinib was associated with a risk of return of lesions, but restart of the treatment rapidly decreased psoriatic inflammation. Retreatment recovery efficacy existed in ~60% of the patients. The reason for this is unknown [4,7,10,42,43,47,48,49,50,51,52]. In conclusion, tofacitinib 5 and 10 mg twice daily showed clinically relevant efficacy versus placebo over a 16-week period [42,43].

#### 1.4.2. OPT Compare—Phase III Studies of Tofacitinib Treatment 

Another phase III trial was OPT Compare. It was conducted to compare tofacitinib 5 mg twice daily or 10 mg twice daily with etanercept 50 mg twice weekly and placebo. It was a randomized multicenter study that proved that the efficacy of tofacitinib 10 mg twice daily is non-inferior at week 12 to the efficacy of etanercept 50 mg twice weekly in psoriasis. The primary end point was evaluated at week 12. Only adult patients with chronic stable plaque psoriasis (for ≥12 months) participated in this trial. The patients were recruited from 122 investigational dermatology centuries from different countries. They were candidates for phototherapy or systemic treatment. The inclusion criteria were a Psoriasis Area and Severity Index (PASI) score ≥12, a Physician’s Global Assessment (PGA) of moderate or severe, and no response to at least one conventional systemic therapy or a contraindication or intolerance to this therapy [7,13]. 

Between November 2010 and September 2012, 1106 patients were grouped in a proportion of 3:3:3:1. In the first group, the patients received 5mg of tofacitinib twice per day, in the second—10 mg twice daily, in the third—50 mg of etanercept twice a week and in the last group—placebo. In this trial, PASI75 was achieved at week 12 by 39.5% patients of the first group, 63.6% of the second group, 58.8% of the third group and 5.6% of the group with placebo. The PGA was better in 47.1% of patients in the first group, in 68.2% in the second, in 66.3% in the third group and in 15.0% in the placebo group. All active groups achieved a Dermatology Life Quality Index score of 0 or 1 in significantly higher percentages compared with placebo (*p* < 0.0001, for all comparisons). The 10 mg tofacitinib-treated group achieved an Itch Severity Item score of 0 or 1 in a greater percentage of patients compared with etanercept, from week 2 up until week 12 (*p* < 0.05 for all comparisons) [14,20,44,53]. Improvement in nail psoriasis, as assessed by the Nail Psoriasis Severity Index score, was also observed during treatment with tofacinitib (5 or 10 mg daily) at week 16 and was generally maintained until week 52 [3,42,47,53,54]. Number of adverse events was similar in all four groups [53].

#### 1.4.3. Adverse Events of Tofacitinib

The adverse events of tofacitinib included skin infections, skin malignancy and cancers of prostate, lungs, breast and pancreas, lymphomas and lymphoproliferative disorders, infections of respiratory system and urinary tract, activation of latent tuberculosis and reactivation of hepatitis B infection, opportunistic infection, pulmonary cryptococcosis, histoplasmosis, gastrointestinal perforations and obstruction. The laboratory adverse events included decreased hemoglobin levels, RBC, neutrophil and lymphocyte count, and elevation of SGPT, SGOT, CPK, HDL, LDL, TG and cholesterol levels. There was urticaria, angioedema, rash, headache, polyneuropathy and hypertension observed in certain examples [11]. 

During phase III studies (tofacitinib 5 and 10 mg), 10–15% patients with active psoriasis arthritis were observed to have increased lipid levels. These changes were dose-dependent. The highest fluctuations were related to HDL, LDL and total cholesterol [50,55,56,57]. Hypertriglyceridemia and metabolic syndrome were higher in patients with psoriasis arthritis than in patients with rheumatoid arthritis treated by tofacitinib [50,58,59]. Studies showed that tofacitinib does not increase cardiovascular disease risk. Similar results were observed in studies with secuckinumab and ustekinumab [41,50,54,60,61,62,63]. During clinical trials estimating the safety of tofacitinib taken 5 or 10 mg twice daily compared with a TNF inhibitor in patients with rheumatoid arthritis, increased risks of pulmonary embolism and mortality in patients who received tofacitinib 10 mg twice daily were noticed [14,64,65]. These symptoms were also observed during another independent study that compared tofacitinib with TNF inhibitors [14,66].

During trials PIVOTAL 1 and PIVOTAL 2 in the period to week 16, both doses of tofacitinib were well tolerated. In approximately 900 patients per study, the rates of adverse events were low and similar in all groups of patients. Nausea, headache and diarrhea rates were mildly elevated compared with placebo. There were no opportunistic infections and gastrointestinal perforations. The risk of infection during taking tofacitinib was similar to that of treatments with another biologics [23,24,42,43,67]. It was observed that tofacitinib increased the risk of herpes zoster virus infection comparatively to placebo [14,68]. Three patients among 363 treated by 5 mg and five patients among 360 patients treated by 10 mg reported herpes zoster in OPT PIVOTAL 1. In OPT PIVOTAL 2, there were three patients among 382 patients treated by 5 mg and one among 381 patients treated by 10 mg. All these infections were mild or moderate. Three patients discontinued the study due to herpes zoster events. There was one case of genital herpes in OPT PIVOTAL 1 (10 mg twice daily) and none in OPT PIVOTAL 2. During trials, there were no cases of tuberculosis or other opportunistic infection, no evidence of multidermatomal (more than two dermatomes) or systemic herpes zoster and also no Cytomegalovirus and Epstein–Barr infections [14,42,69]. 

The most frequent infections were nasopharyngitis, which occurred in OPT PIVOTAL 1, occurring in 5.5% of patients treated with 5 mg tofacitinib, 8.6% patients treated with 10 mg tofacitinib, and 11.3% with placebo. In OPT PIVOTAL 2, it occurred in 8.4% patients treated with 5 mg tofacitinib, 7.9% patients treated with 10 mg tofacitinib, and 5.6% with placebo. Quantity of diarrhea (2.2–4.5%) and headache (4.2–6.9%) were higher with tofacitinib than placebo (0–1.7% and 2.8–3.1%, respectively). Incidence of nausea during taking of tofacitinib was similar to placebo (0.5–2.8%) [43].

During the first 16 weeks of research, there were four patients with tumors (excluding nonmelanoma skin cancer) in OPT PIVOTAL 1 (malignant melanoma, malignant melanoma, esophageal carcinoma, prostate cancer) and none in OPT PIVOTAL 2. There was one case of basal cell carcinoma and one case of squamous cell carcinoma (10 mg twice daily) in OPT PIVOTAL 2 [42,43].

In a study with tofacitinib levels of HDL cholesterol, LDL cholesterol and triglycerides were higher during 4 week observations. In the next period (from 4th to 16th week), the levels were stable. It was not connected with increases in cardiovascular risk. Major adverse cardiovascular cases were reported in two patients receiving tofacitinib 5 mg twice daily, one receiving 10 mg twice daily and none with placebo; all cases were unrelated to the treatment by tofacitinib [14,43,69].

Higher levels of median cholesterol and creatinine phosphokinase (CPK) and lower levels of median hemoglobin were confirmed with tofacitinib during OPT PIVOTAL 1 and OPT PIVOTAL 2. Seven patients had a CPK level of >10 times the upper limit of normal. Among these patients, there were observed moderate myalgia, mild neck pain, and mild arthralgia. No rhabdomyolysis was reported. Mild decreases of blood lymphocyte and hemoglobin were reported in patients with psoriasis healed by tofacitinib; however, these changes decreased and were usually reversible. No severe anemia was confirmed [14,65,70].

### 1.5. Baricitinib—General Information and Clinical Trials 

Baricitinib selectively inhibits JAK1/JAK2 tyrosine kinases [71]. Baricitinib has also been tested in clinical double-blind, placebo-controlled, dose-ranging phase 2b studies [4,45].

Before described studies, patients were qualified to be candidates for phototherapy or systemic therapy. Inclusion criteria were: age ≥18 years old, chronic plaque psoriasis for ≥6 months, ≥12% of body surface involved with psoriatic lesions, PASI scores of ≥12 and static Physician’s Global Assessment (sPGA) scores of ≥3 on a 6-point scale at study entry. Exclusion criteria were history of serious infections or illnesses, active infections, serious comorbid cardiac or hepatic conditions, immunocompromised states, previous treatment with an oral JAK inhibitor, treatment with a biologic agent or monoclonal antibody within 8 weeks before study, treatment with systemic psoriasis therapy or phototherapy within 4 weeks before study and topical psoriasis therapy within 2 weeks before study.

Patients were randomized to receive placebo or oral baricitinib at 2, 4, 8 or 10 mg once daily for 12 weeks [71]. In this 12-week dose-ranging study, encouraging results in treatment were noticed [13]. The primary end point was Psoriasis Area and Severity Index (PASI) 75% (PASI-75) at 12 weeks. A 75% reduction in PASI was achieved by 43% patients treated with baricitinib 8 mg once daily and 54% treated with 10 mg versus placebo group (17%) [7]. Patients achieved significantly higher PASI75 response rates at week 12 compared with placebo. The majority (more than 81%) of the respondents maintained their scores through week 24 [45,71].

In conclusion, patients with moderate to severe psoriasis treated with baricitinib for 12 weeks obtained significant improvements in PASI-75 rates versus patients treated with placebo [71].

#### Adverse Effects of Baricitinib

There were no serious side effects observed for baricitinib, and this medicine was well tolerated during trial; however, changes in laboratory parameters were similar to those reported for tofacitinib. Baricitinib was observed to cause small dose-related decreases in neutrophil count and hemoglobin levels, as well as small increases in creatinine and lipoprotein levels [4,14,52,72,73]. Opportunistic infections were not observed in any treatment group [71].

### 1.6. Ruxolitinib—General Information and Clinical Trials

Ruxolitinib is a JAK1/JAK2 inhibitor that blocks signal transduction of multiple proinflammatory cytokines [69,72]. This JAKs inhibitor was used as a topical treatment. 

The topical ruxolitinib cream was checked during three psoriasis clinical trials. In a phase 2 vehicle-controlled study in mild and moderate psoriasis, ruxolitinib reported PASI reduction, although no clear dose–response was observed [13].

During the next trial, a double-blind study, ruxolitinib in 1.0% or 0.5% cream used once per day or 1.5% cream twice per day was compared to two active comparators: calcipotriene 0.005% cream and betamethasone dipropionate 0.05% cream for 28 days [13,69]. Ruxolitinib achieved clinical efficacy and was non-inferior to active comparators. One percent ruxolitinib cream as well as 1.5% cream improved erythema, scaling, lesion thickness, erythema and reduced lesion area. It caused their composite lesion score to decrease by more than 50% compared with 32% for active comparators [69,72].

Finally, a third study conducted in 25 patients showed that epidermal hyperplasia was reduced with ruxolitinib in most patients [7]. Inclusion criteria in this study were: limited psoriasis (covering <20% of the body surface area) and age 18–65 years. Psoriatic lesions were rated on a scale of 0–4 for erythema, thickness and scaling. Disease activity in each patient was also scored by Physician’s Global Assessment scale. The biopsies of pretreatment and posttreatment skin were compared with healthy skin. In these biopsies, histopathology, immunohistochemistry and mRNA expression were evaluated. Laboratory parameters were also measured: ruxolitinib concentrations in plasma, cytokine stimulated phosphorylated signal transducer and activator of transcription 3 phosphorylation (pSTAT3) levels in peripheral blood cells [71]. Topical ruxilitinib phosphate 1.0% or 1.5% cream was used once or twice daily for 28 days to 2–20% body surface area in five sequential groups of patients, each consisting of five patients [69,72]. After application of ruxolitinib phosphate cream 1.0% and 1.5%, there was significant improvement in lesion scores [72]. During the study, these were observed: decreased dermal inflammation, reduction of epidermal hyperplasia, reduction of dermal inflammation, downregulate transcription of Th1 and Th17 cytokines in psoriatic skin lesions and also reduction of CD3, CD11c, Ki67 and keratin 16 observed during immunohistochemical analysis. There were notable interconnections between clinical improvement and decreases in markers of Th17 lymphocyte activation, epidermal hyperplasia and dendritic-cell activation [4,45,69,72,74]. However, it was not a sustained improvement after discontinuation [54].

In conclusion of this study, topical ruxolitinib is pharmacologically active in patients with active psoriatic lesions and modulates proinflammatory cytokines [69,72].

### 1.7. Adverse Events of Ruxolitinib

During the double-blind study when ruxolitinib 1.0% or 0.5% cream once per day or 1.5% cream twice per day was compared to two active comparators, inhibition of phosphorylated STAT3 in peripheral blood cells was not observed, suggesting limited systemic exposure [7,14]. Systemic absorption was minimal, and there was no evidence of systemic toxicity [75]. Topical ruxolitinib was found to be well tolerated, safe, and efficacious in short-term treatment in a smaller cohort of patients [9].

During topical application in the 25 patients, there was no noticeable inhibition of pSTAT3 in peripheral blood cells observed. It was relevant to be consistent for low steady-state plasma concentrations of ruxolitinib [69,72].

### 1.8. Filgotinib—General Information and Clinical Trial

Filgotinib is an oral selective JAK1 inhibitor. The clinical studies of filgotinib in psoriatic arthritis patients and in other illnesses including rheumatoid arthritis, ankylosing spondylitis and ulcerative colitis are still undergoing and have not been confirmed for selling yet [76].

A randomized, double-blind, placebo-controlled phase II trial (EQUATOR) was conducted in active moderate-to-severe psoriasis arthritis. During these studies, evaluating the efficacy and safety of filgotinib in psoriatic arthritis was assessed [76].

The trial was conducted between 9 March and 27 September 2017. In this study, 191 adult patients from 25 cities in seven countries of Europe (Belgium, Bulgaria, Czech Republic, Estonia, Poland, Spain, and Ukraine) were screened. Of those, 131 patients were randomly divided into treatment regimens: 65 patients for filgotinib in dose 200 mg orally once a day and 66 patients for placebo orally once a day, for 16 weeks [75]. Inclusion criteria were: aged ≥18 years, active moderate-to-severe psoriatic arthritis, documented history or active of plaque psoriasis and an inadequate response or intolerance to at least one conventional synthetic disease-modifying anti-rheumatic drug (csDMARD) [76]. During the study, patients continued to take csDMAR= if they had received this treatment for at least 12 weeks before screening and had been taking at the same dose for at least 28 days before study [75].

The primary endpoint was proportionate to the patients who achieved 20% improvement in the American College of Rheumatology response criteria (ACR20) at week 16 [75]. Filgotinib showed better efficacy in the ACR20 and ACR50 rates at week 16 versus placebo. Filgotinib group achieved ACR20 in 80%, ACR50 in 55%, LDA (DAPSA ≤ 14) in 49%, and PASI75 in 45% of patients. The percentages of the placebo group were respectively 33%, 12%, 15%, and 15% [29,76]. The development in nail psoriasis at week 16 did not achieve statistical significance, probably because of the short study duration and relatively small amount of patients with nail psoriasis [75,76]. In total, 92% patients receiving filgotinib and 97% patients receiving placebo finished the study [75].

#### Adverse Events of Filgotinib

During the EQUATOR study, good tolerance of filgotinib was observed. The incidence of adverse events including infections that required treatment was similar in filgotinib group versus placebo group at 16 weeks (57% versus 59%). Most of adverse events were mild or moderate.

The most frequent adverse events were headache and nasopharyngitis (similar amount in both group of patients). There were no cases of thromboembolic events, malignances or opportunistic infections, and only one case of herpes zoster infection was observed. One serious treatment-emergent adverse event of pneumonia was reported in the filgotinib group. A decrease of platelets, and increases of hemoglobin, HDL and lymphocyte counts were observed in the filgotinib group [75,76].

### 1.9. Decernotinib—General Information and Clinical Trial

Decernotinib is the selective inhibitor of JAK3. In first evaluations, it was shown that it can modulate proinflammatory responses of autoimmune diseases such as rheumatoid arthritis. 

During placebo-controlled monotherapy study, decernotinib used in doses 50–150 mg twice per day improved clinical signs of rheumatoid arthritis. Later, during two phase II studies, decernotinib was combined with methotreksat and also improved the symptoms of rheumatoid arthritis compared with placebo [4,46].

#### Adverse Events of Decernotinib

Different adverse effects were noticed during these researches: infections—two herpes zoster infections and one case of tuberculosis, neutropenia—in patients in the methotrexate study, increases of liver transaminase, creatin and lipid levels. The metabolite of decernotinib is a potent inhibitor of cytochrome P450, which is involved in metabolism of different drugs. This interaction can complicate the use of decernotinib [4,46].

## 2. Conclusions

The choice of treatment in psoriasis depends on the severity of the disease assessed on the available scales. The assessment considers the extent of the lesions, their locations and severity, the response to previously applied treatment and the impact on the quality of life of patients. Definitions of disease severity are mainly based on the criteria for including patients in randomized controlled trials. Although the classification of disease severity varies, mild psoriasis is generally characterized as a disease that can be treated locally. In moderate or severe psoriasis, an escalation of treatment using phototherapy or a systemic drug can be necessary [77]. From the available treatment options, in the first line are topical steroids, topical vitamin D analogues, retinoids, hydroxyurea and fumaric acid esters. During topical treatment, it is important to use creams with urea, salicylic acid, and cignolin. More advanced external treatment includes UVB or psoralen plus UVA phototherapy. Patients with severe psoriasis can be treated with systemic medications such as methotrexate, cyclosporine and acitretin [78]. Unfortunately, the effectiveness of these drugs is often insufficient and they can cause a variety of side effects. Currently, biologic drugs are an important therapeutic option. The decision to use biologic agents must be carefully considered, based on the clinic and the individual patient risk profile. The type of biologic for psoriasis treatment is chosen according to disease severity and comorbidities. A history of previous biologic treatment and its effectiveness are also important. The main indication for biologic treatment is “moderate-to-severe” psoriasis, but the practicing clinician needs to consider what the exact severity is before qualifying the patient for the treatment. The European Medicines Agency (EMA) guidelines refer to indications such as: failure of topical therapies to control the disease; body surface area (BSA) involvement >10% or PASI 10 to 20; thick lesions located in difficult-to-treat regions with BSA involvement <10% may also be considered; and category “moderate to severe” on the PGA (Physician’s Global Assessment). The NICE recommendations for disease assessment state that both disease severity and impact are relevant and include the use of indexes such as PASI, PGA, patient assessment, enquiry about difficult-to-treat sites, NAPSI (Nails Psoriasis Severity Index), in which nails are the primary indication for systemic therapy, DLQI (Dermatology Life Quality Index) and assessment of anxiety and depression [79,80]. In addition to the excellent therapeutic effects of biological drugs in psoriasis, there is more talk about the loss of efficacy and its causes. The main cause is the induction of an immune response directed against the foreign protein molecules. Consequently, antibodies directed against the drugs (ADA) are produced. The presence of ADA is associated with lower serum drug levels and loss of clinical efficacy. Furthermore, an increased incidence of ADA-related adverse drug reactions is observed [81]. The development of ADA in psoriasis is still uncertain, but it seems to be similar to the presence of ADA during biologic treatment in other autoimmune diseases such as Crohn’s disease and rheumatoid arthritis. Strand et al. [82], in a systematic review based on data from published reports, found that 50% of patients receiving adalimumab and infliximab developed ADA. Certain factors may influence the immunogenic potential of the agents. These may include the molecular structure of the biologics, concomitant use of methotrexate or other immunosuppressive/anti-proliferative agents, dosage and regimen of the biologic administered and a history of ADA with previous biologic treatment. In addition, patient-related factors may include sex, ethnicity and comorbid conditions [82]. Previous studies indicate well-documented safety and tolerability of biological drugs used in psoriasis. General adverse events (AEs) of biologic treatment are similar. The most frequent (>10%) are various infections such as upper and lower respiratory tract infections, rhinitis, sinusitis, pharyngitis and nasopharyngitis. Serious AEs are rare (<1%) and may include sepsis, viral reactivation (VZV, HBV, HSV), tuberculosis reactivation and fungal infections. Compared to treatment of psoriasis with non-biologic therapy, biologic therapy has not been significantly associated with major adverse events such as cardiovascular events, malignancy, or death beyond what is anticipated in the overall psoriasis population. Other AEs associated with the liver, including severe hepatic reactions, hepatitis, cholestasis and acute liver dysfunction have been reported. Pancytopenia and aplastic anemia were observed rarely during TNF-α inhibitor treatment. In addition, several cutaneous adverse reactions have been associated with anti-TNF drugs. These include eczematous dermatitis, lupus-like skin reactions, leucocytoclastic vasculitis, lichen planus, lichen-planus-like eruptions and alopecia. The safety profile of anti–IL-12/23 has been reported from the results of large clinical trials, including PHOENIX 1, PHOENIX 2 and ACCEPT. The most common AEs were infections, while 0.7% of patients had a cardiac disorder and 0.7% had a serious infection. The most common adverse events that occurred during anti–IL-17A therapy were infections, injection site reactions, nausea and neutropenia [81]. The frequency of adverse effects during therapy with JAK inhibitors is similar to that of other biologic drugs. JAK inhibitors can inhibit the activity of many cytokines that play a role in the pathogenesis of psoriasis. Therefore, JAK inhibition may be associated with an increased risk of infections [83]. Studies to date do not indicate that JAK inhibitors are superior to recent biologic drugs in terms of efficacy. However, the efficacy observed for JAK inhibitors is better than for some currently used systemic therapies, such as some older biologic drugs such as etanercept [15]. JAK inhibitors, due to their lack of increased incidence of side effects compared to other biologic drugs, can be included in the psoriasis treatment algorithm because they are oral and less expensive than modern biologic drugs [15].

The expected results from the clinical trials about JAK inhibitors will be a major step toward extending the therapeutic spectrum of psoriasis by oral compounds. Currently, the number of registered studies on JAK inhibitors in psoriasis is rapidly growing [9,13]. The well-established efficacy of JAK inhibitors in inflammatory disorders, particularly rheumatoid arthritis and ulcerative colitis, suggests the potential of their positive effects in a myriad of inflammatory dermatoses as well [8]. More selective JAK inhibitors are currently in clinical trials [9]. Based on the experience with tofacitinib, numerous JAK inhibitors are tested as oral drugs or as topical formulation for psoriasis. Thus far, the efficacy of topical JAK inhibitors for psoriasis is not convincing [13]. Nevertheless, further studies are needed to evaluate long-term treatment effects with these drugs. 

## Data Availability

Not applicable.
